# Maintaining information about speech input during accent adaptation

**DOI:** 10.1371/journal.pone.0199358

**Published:** 2018-08-07

**Authors:** Zachary Burchill, Linda Liu, T. Florian Jaeger

**Affiliations:** 1 Department of Brain and Cognitive Sciences, University of Rochester, Rochester, New York, United States of America; 2 Department of Computer Science, University of Rochester, Rochester, New York, United States of America; Universidad de Salamanca, SPAIN

## Abstract

Speech understanding can be thought of as inferring progressively more abstract representations from a rapidly unfolding signal. One common view of this process holds that lower-level information is discarded as soon as higher-level units have been inferred. However, there is evidence that subcategorical information about speech percepts is not immediately discarded, but is maintained past word boundaries and integrated with subsequent input. Previous evidence for such subcategorical information maintenance has come from paradigms that lack many of the demands typical to everyday language use. We ask whether information maintenance is also possible under more typical constraints, and in particular whether it can facilitate accent adaptation. In a web-based paradigm, participants listened to isolated foreign-accented words in one of three conditions: subtitles were displayed concurrently with the speech, after speech offset, or not displayed at all. The delays between speech offset and subtitle presentation were manipulated. In a subsequent test phase, participants then transcribed novel words in the same accent without the aid of subtitles. We find that subtitles facilitate accent adaptation, even when displayed with a 6 second delay. Listeners thus maintained subcategorical information for sufficiently long to allow it to benefit adaptation. We close by discussing what type of information listeners maintain—subcategorical phonetic information, or just uncertainty about speech categories.

## Introduction

Speech understanding can be thought of as inferring progressively more abstract linguistic representations, such as phonemes, words, meanings, etc., from a rapidly unfolding signal. One common view is that this abstraction process is accompanied by complete and immediate *compression*, whereby information about lower-level representations is discarded as soon as a higher-level unit has been inferred. The motivation underlying this view is that attention and memory resources are strongly bounded, so that the high-dimensional perceptual signal *needs* to be simplified immediately [1] (see also [2]; for discussion, see [3]). An intuitive example in line with this immediate compression view is the experience of having difficulty recalling the exact word sequences used in a past conversation, even when one recalls its content.

Beyond such intuitive examples, the immediate compression view was motivated in part by the early finding that listeners rapidly categorize the speech signal into invariant phonological categories (categorical perception [4]), suggesting that subcategorical information was abandoned early during speech perception. Additional support came from early research on "perceptual stores". This work found that memory of detailed perceptual information often decays within fractions of a second [5,6]. Evidence like this shows that information about the original signal is lost as perceptual processing proceeds. It does, however, leave open whether *all* subcategorical information is lost and *when* such information is lost during processing.

Indeed, there are at least two reasons to believe that speech perception is more than just complete and immediate compression. First, there is evidence that at least *some* subcategorical information is maintained long enough to become part of the representation of the word in long-term memory. For example, word recognition is facilitated for words that have previously been heard from the same talker [7–10] (see also [11–15], for review, see [16,17]). This is only possible if subcategorical information somehow is maintained for long enough to become part of long-term memory.

Second, there is now a small but growing body of research suggesting that some auditory information does not decay as rapidly as previously assumed. For example, listeners can maintain certain amounts of fine-grained auditory information about pure tones in memory for up to 10 seconds [18]. At least under experimental conditions, even more complex auditory information such as vowel formants seem to be maintained for at least three seconds [19]. At the speeds typical of conversational speech, three seconds would correspond to approximately 9 to 15 words [20], much longer than immediate compression would suggest. Furthermore, evidence suggests that attentional resources can be directed toward auditory representations maintained in short-term memory (for review, see [21]). For example, Backer & Alain [22] showed that cueing attention to auditory representations even four seconds after stimulus offset enhanced listeners' ability to attenuate change deafness. It seems that attentional resources can even be oriented toward specific features within the auditory representation, such pitch [21]. This suggests that attentional resources could be invested to facilitate maintenance of rich perceptual information.

However, many of these earlier works investigated the limits of auditory memory under conditions hardly representative of every day speech perception. Specifically, these studies typically employed relatively simple speech stimuli presented in isolation (e.g., single vowels [19], syllables [23], for review, see [24]). Crowder [19] tasked participants with deciding if two auditory stimuli presented with varying intervening lag were identical vowels. Typical studies of this variety also involve many repetitions of the same stimulus type. As a consequence, participants knew what information from the speech signal to maintain to successfully complete the task. These studies might thus be more informative about speech perception under these very specific conditions, rather than being reflective of the limits of subcategorical information maintenance during everyday speech perception. As we argue below, similar concerns apply to the interpretation of more recent work, including research on the limits of subcategorical maintenance during sentence understanding [3,25–29].

The goal of the present research is to contribute to the understanding of attentional and memory limitations during speech perception. Specifically, we focus on the perception of—and adaptation to—foreign accented speech. We ask how long after its occurrence in the signal does information remain available to guide accent adaptation? We begin with a summary of previous work and then outline the approach we take to expand on these studies.

### Subcategorical information maintenance during speech perception

A number of studies address the most literal interpretation of the immediate compression view, probing the extent to which subcategorical information can be maintained beyond the segment [25–31] (for a review, see [32]). For example, McMurray et al. [31] had participants view scenes of five objects and click on the object that was spoken aloud. Participants’ eye-movements were tracked, while they listened to and executed these instructions. On critical trials, the scene contained both the target word (e.g., "telephone") and an onset competitor (e.g., "Delaware") which shared the segments following the onset (/t/ or /d/) with the target. Such scenes elicit competitor effects: in addition to the target referent, participants fixate competitor referent more often than other unrelated distractor referents in the scene [33]. In McMurray et al. [31], the target and competitor always differed in whether the onset was a voiced (e.g., /d/ as in "Delaware") or voiceless plosive (e.g., /t/ as in "telephone"). Between trials, McMurray and colleagues manipulated the voice onset time (VOT) of the target word’s onset plosive, making its voicing more or less ambiguous (VOT is the primary cue to voicing in English [34]). Participants exhibited increasingly slower recovery times—the time it took to shift their gaze from the competitor to the target after the point of disambiguation (e.g. the /f/ vs. /w/ in "telephone" and "Delaware")—the more ambiguous the VOT was. If listeners were immediately discarding the (subcategorical) VOT information, they should not have exhibited such within-category differences in eye-movements four-to-five phonemes after the initial phone (for similar results from neuro-imaging, see also [35]).

Another line of studies has found that some subcategorical information can be maintained for longer, even *past word boundaries*. For example, following original work by Connine et al. [28], Bicknell et al. [3] had participants listen to sentences with words in which a contrast was artificially manipulated along the voicing continuum (e.g., from "tent" to "dent") to create more or less ambiguous "(d/t)ent" instances. In all target sentences these manipulated words were followed by disambiguating contexts (e.g., either "There’s a (d/t)ent in the fender", biasing towards "dent", or "There’s a (d/t)ent in the forest", biasing towards "tent"). The disambiguating right-context occurred either 3 syllables or 6–8 syllables after the manipulated word. After each sentence, participants answered which word they heard (e.g. "tent" or "dent"). Participants' answers were affected by both the phonetic information (e.g., the VOT of the initial phone of the target word) and the disambiguating right-context (whether the sentence continued with "fender" or "forest"). Similar results were obtained in other experiments [27,29]. Connine et al. [28] reported that subcategorical information is maintained for three but not six to eight syllables. However, Bicknell et al. [25] pointed to a procedural problem in Connine et al.’s study. Once this problem was removed, Bicknell and colleagues found no evidence of decay in the maintenance of subcategorical information even at the longest lag tested, 6–8 syllables).

In all these studies, the contrast that participants were asked to categorize remained constant across the entire experiment. Furthermore, the repeated critical segment always occurred in a predictable location in the target sentences in each study. For example, in the second experiment of Szostak & Pitt [29], participants judged whether they heard /s/, as in "sip", or /ʃ/, as in "ship", in the target word. The /s/-/ʃ/ contrast was the only one participants needed to keep track of, and all sentences began with "The (?sʃ)ip was …". Thus participants knew *what* aspect of the signal to maintain (e.g. the /s/-/ʃ/ contrast) and *when* that aspect appeared in the signal (e.g. at the onset of the second word). It is possible, if not likely, that this made the task much easier: participants could in principle improve performance by only maintaining information about a specific small part of the signal. Thus, these studies leave open whether subcategorical information is maintained past word boundaries under circumstances that more closely resemble everyday speech processing.

This is question we seek to address here. In order to approach questions about the limits of maintaining information under task demands that more closely resemble everyday speech perception, we explore a paradigm novel for the study of uncertainty/information maintenance during speech perception. This paradigm draws on another line of work, which we introduce next: research on adaptation to an unfamiliar foreign-accent. As we detail in the discussion, the paradigm we employ—and the results we obtain—also speak to another pressing question about information maintenance: the nature of the maintained information. Specifically, we will discuss how our result suggest that listeners can maintain at least some aspects of information—at the *phonetic* level or below—for much longer than previously assumed (rather than to just maintain degrees of uncertainty, cf. [3] for discussion).

### Using foreign-accents to study information maintenance

Understanding an unfamiliarly accented talker can be difficult initially [36], but listeners tend to get better with exposure to the talker [37,38]. This process, sometimes referred to as *accent adaptation*, can be facilitated by context: knowing what word was intended does not only facilitate recognition of that word [39], but can also lead to better recognition of subsequent materials [40] (for similar work with degraded speech, see [41]). For example, Mitterer & McQueen [40] exposed native Dutch listeners to excerpts of Australian and Scottish English—either accompanied by subtitles or not. During a test phase, listeners had to transcribe other audio excerpts from the same English variety (in the absence of subtitles). Participants who had been exposed to subtitled speech performed significantly better at the transcription task, compared to listeners who had not received subtitles during exposure. This shows that labeling information provided by the context (in this case, subtitles) can facilitate adaptation to regional varieties of English (at least for L2 listeners).

In Mitterer & McQueen [40], subtitles were displayed simultaneously with the accented speech, similar to standard subtitling in movies. Here we adapt this paradigm to our goals. We expose listeners to foreign-accented speech, while subtitles are displayed either simultaneously, at various delays, or not at all. During a later test phase—where speech from the same foreign-accented talker is now presented without subtitles—we then assess how well listeners have adapted to the accented speech. By comparing performance during the test phase, we can assess the effect of subtitle timing during exposure. Research with a similar paradigm has been used to study how contextual information enhances speech comprehension in the moment: Sohoglu et al. [42] manipulated the timing of subtitles in relation to acoustically degraded speech to determine the delay at which the enhancement in perceived clarity of speech from the contextual information would begin to decline.

In this paradigm, the subtitles thus parallel the role of right-context in the previous work discussed above [3,25–29]. As we detail next, this paradigm allows us to test whether information about the speech signal is maintained, and for how long. Specifically, the paradigm we explore here allows us to address the questions we highlighted above.

Accented speech exhibits high-dimensional deviations from familiar speech, and optimal adaptation to an accented talker would require learning the nature of these deviations. In our paradigm listeners, who are specifically selected as being unfamiliar with the particular foreign accent, do not *a priori* know exactly what phonetic information is critical for adaptation. And being unfamiliar with this accent, listeners do not know *where* in the signal relevant phonetic information will occur, or *what* type of information that will be. This makes the task demands of the present paradigm somewhat more similar to everyday speech perception, while still allowing control over the relative timing of labeling information.

Using this paradigm, we present two web-based experiments on accent adaptation. Experiment 1 serves two aims. First, we test whether adaptation to accented speech is facilitated when listeners are exposed to subtitled accented speech, compared to exposure to accented speech without subtitles. Like previous work [40,42,43] this first manipulation uses subtitles that are presented concurrently with the speech input. Second and key to the current study is the question of whether subtitles that are displayed after the speech input—thereby constituting right-context—facilitate accent adaptation. If so, this would suggest that listeners can maintain information past word boundaries and integrate this information with later context for learning (in the general discussion, we discuss alternative explanations and why we do not think that our data supports them). These results would imply that the speech signal is not immediately compressed and discarded, contrary to the extreme compression view.

To anticipate our results, Experiment 1 finds that subtitles provide a small but significant facilitatory effect for accent adaptation. Critically, statistically identical facilitatory effects are also observed when the subtitles are displayed after the speech input. Encouraged by this result, we conducted an additional experiment to explore the temporal limits of such maintenance. In order to test for how long information relevant to accent adaptation can be maintained perfectly, Experiment 2 manipulates the amount of time that passes between the presentation of the speech stimulus and the presentation of the subtitles (right-context).

## Experiment 1

In an exposure-test paradigm, participants listened and responded to speech produced by a Spanish-accented talker. In the previous work that inspired us to use a subtitle-based paradigm [40], participants watched a 30-minute video of accented talkers in a commercial television show or a film. We adapt this paradigm for the study of information maintenance, which requires control over the timing of subtitles relative to the speech input. We thus present small chunks of speech input. In the following experiments, we present isolated words.

Each participant was randomly assigned to one of three between-participant conditions that manipulated the use of subtitles during the **exposure phase**. Participants in the Absent condition received speech input (which was one word per trial) without any subtitles. Participants the Concurrent subtitle condition saw the subtitle for each word presented simultaneously with the speech input. Finally, participants in the Delayed condition saw the subtitle immediately *after* the end of the speech input. In the **test phase**, we use transcription accuracy as a measure of accent adaptation (following [37]). During test, participants in all three conditions heard novel isolated words produced by the same talker they heard during exposure (but without subtitles), and transcribed the word they heard for each of these trials.

### Methods

#### Participants

Experiment 1 and 2 aimed for 60 successful participants per condition (see Exclusions below). To this end, 245 participants were recruited using Amazon's Mechanical Turk (www.mturk.com), with the human subject research for both experiments being approved by the University of Rochester's Research Subjects Review Board. Recruitment asked for monolingual talkers of English. Participants were randomly assigned to one of the three subtitle conditions.

#### Procedure

The experiment was conducted over the web with Mechanical Turk. The experiment consisted of four phases: it began with a practice phase, followed by an exposure phase, then a test phase, and finally a survey.

**Fig 1:**
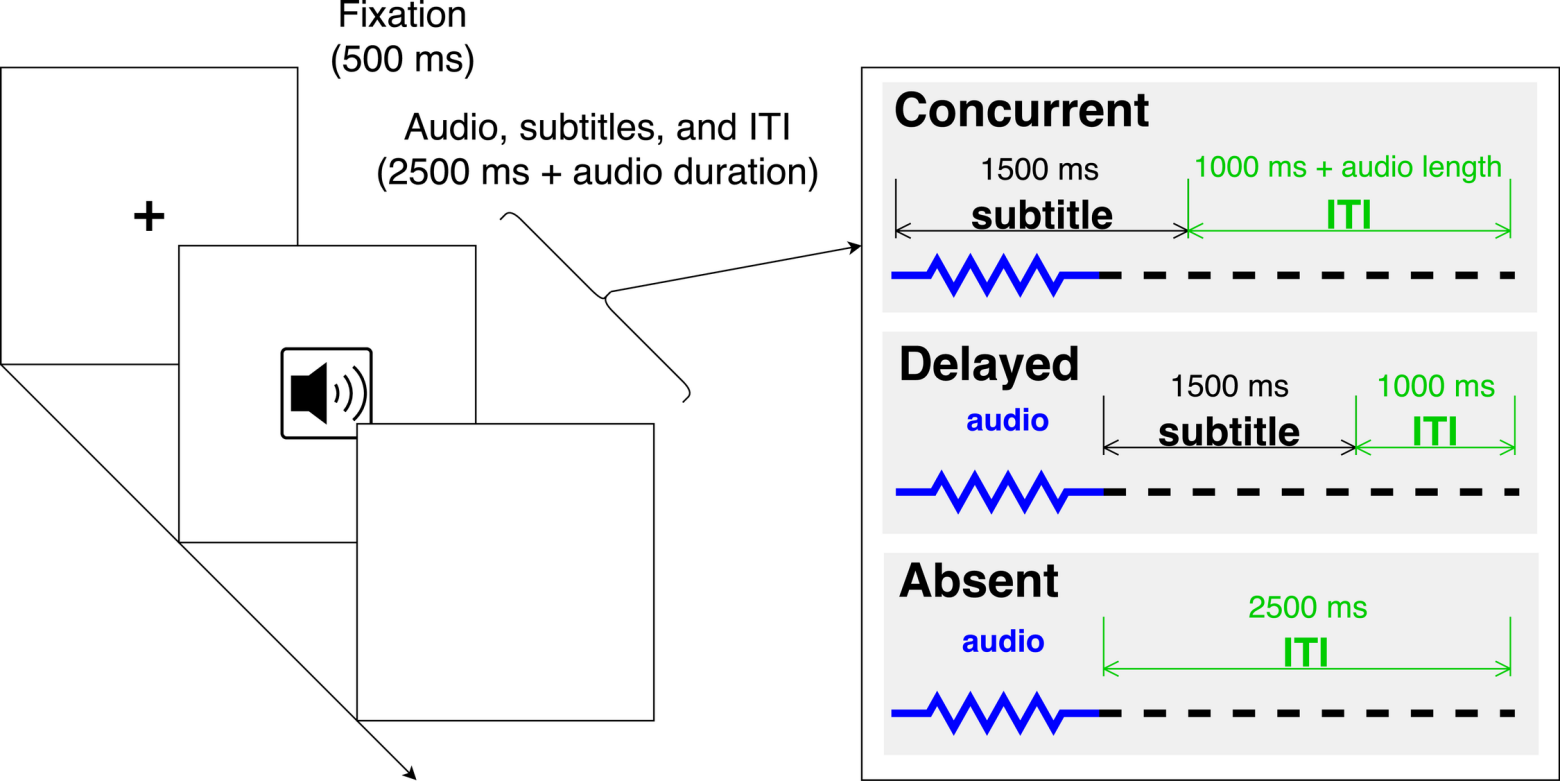
Schematic illustration of exposure phase trial structure. The audio icon on the image represents the beginning of the audio; no such icon was visually displayed to participants.

After four practice trials, participants went through the **exposure phase**, which consisted of 80 experimental trials. The structure of the exposure trials is illustrated in Fig 1. Each trial started with a fixation cross that was displayed for 500 milliseconds (ms). The speech input started playing immediately afterward. In the Concurrent condition, the subtitle was displayed for 1500 ms, starting concurrently with the speech input (Fig 1, top-right). In the Delayed condition, subtitles were also displayed for 1500 ms, but immediately following the offset of the speech input (Fig 1, middle-right). The duration for which the subtitles were displayed was thus held constant across the Concurrent and Delayed conditions. In the Absent condition, no subtitles were displayed (Fig 1, bottom-right). The total duration of each trial was held constant across conditions: the next trial always began 2500 ms after the offset of the speech input. When no subtitles were displayed, the screen was blank (labeled "ITI" in Fig 1).

To measure whether participants were actively engaged in the exposure phase, 12 catch trials were distributed throughout exposure. These trials were identical to other trials, but a pure tone was played briefly before the beginning of these trials. Participants were instructed to press the space bar when they heard this tone (participants were forced to repeat the practice block until they correctly responded to the two additional catch trials that were in that block). We recorded the number of false positives (pressing the space bar when no tone was played) and false negatives (failure to press the space bar during catch trials) during exposure.

During the **test phase** in all conditions, each trial began with the accented talker saying the word. Participants then transcribed the word to the best of their ability in a text box and pressed the enter key to move on to the next trial. The test phase consisted of 40 such trials. Regardless of the exposure condition, test trials never contained subtitles.

After the test phase, participants took a short **exit survey** that asked about their language background and ethnicity, audio quality, and what type of audio equipment they were using. For a complete lists of survey questions, see S1 Questionnaire.

#### Materials

The 120 monosyllabic CVC words participants listened to during exposure and testing were produced in isolation by a Spanish-accented female talker and the recordings were taken from the Hoosier Database of Native and Nonnative Speech for Children [44]. These particular words were chosen as they represented the 120 least comprehensible words produced by the talker, as measured by a separate pre-test norming experiment also conducted over Amazon's Mechanical Turk. The mean amplitude of all clips was adjusted to 70 dB.

The stimuli for the four practice trials consisted of recordings of isolated monosyllabic words spoken by a Japanese-accented female talker taken from the same corpus (but included CVC and more complex words).

The 120 Spanish-accented audio clips were divided into three blocks of 40 words in such a way that the types of onsets, vowels, and codas were roughly equal across blocks. Using a Latin-square design, we rotated which block served as test and which two blocks served as exposure across participants so that each block served as test to equal numbers of participants in each condition. We also reversed the internal order of all block across participants so that each block order and block-internal order was heard an equal number of times in each condition. The block structure for the first two blocks (exposure) was opaque to participants. For the third block (test), the task changed from passive listening to transcription.

#### Scoring

All transcriptions were automatically scored for accuracy. A transcription was counted as "correct" if it matched the spoken word or matched an existing homophone of the word. After a subset of automatically processed transcriptions was found to be highly accurate by manual review, all transcriptions were processed automatically.

#### Exclusions

Sixty-nine participants were excluded from Experiment 1 (see Table 1). Although participants were told beforehand that they were required to be monolingual speakers of American English, a number of participants reported on the post-test survey that they had not met those requirements and were therefore excluded. Since the purpose of the current study is to investigate maintenance under more naturalistic constraints, i.e., when participants do not know *a priori* what aspects of the signal to maintain and where in the signal these parts will occur, participants likely to be familiar with similar accents (those who reported that they had family members with Hispanic backgrounds or those who reported familiarity with equally strong foreign accents) were excluded from the analyses.

**Table 1:** Participant exclusions in Experiment 1. Some participants were excluded for multiple reasons.

Total participants:	245	100%
Reason for exclusion	n	%
Participant's family likely to include Spanish speakers	20	(8%)
Participant not monolingual	47	(19%)
Participant reported familiarity with strong foreign accents	6	(2%)
Used computer speakers or unknown audio quality	6	(2%)
Client-side computing error	1	(0%)
Failed >4 catch trials (out of 12)	7	(3%)
Outliers in transcription performance	2	(1%)
Participants remaining	176	(72%)

Because the current study was run over the web, audio quality and audio equipment varied from participant to participant. Previous studies have demonstrated that despite this variability, web-based experiments on speech perception are feasible [45–47]. This includes studies on accent adaptation [48–50]. To reduce the between-speaker variability, we required participants to use either in-ear or over-ear headphones when taking the experiment. Participants who did not comply with these restrictions (based on their response in the exit survey) were excluded. Participants who made more than four catch trial errors or who had experienced technical difficulties were also excluded. After these exclusions, we excluded any participants whose mean transcription accuracy during test was more than three standard deviations from the overall mean of all remaining participants. This left 62 participants in the Absent condition, 57 in the Concurrent condition, and 57 in the Delayed condition.

#### Determining which items benefit from subtitles

Here we are interested in whether (and when) delayed subtitles facilitate accent adaptation. For this reason, test items that do not benefit from exposure to concurrent subtitles, compared to exposure without subtitles, are not informative for the present purpose. This issue, its reasons, and its consequences occurred to us only after all experiments had already been conducted. Here we describe how we decided to address it.

There are several reasons why a test item might not benefit from subtitling during exposure. One important reason for the present purpose is that the test item might not have benefitted from *any* exposure (subtitled or not). For example, an item might contain a speech error or other accent-unrelated problem that leads participants to misunderstand it. An item—recall that items were isolated words—might also contain accent features that, even if perfectly learned, do not necessarily lead to improved transcription. For example, the /ɪ/ in "sip" is not a phoneme in Spanish, and L2 English learners often pronounce it as /i/, pronouncing "sip" as "seep". Even a listener who has learned this only has a 50% chance of determining if /sip/ referred to "sip" or "seep" (provided that all other phonemes were recognized accurately). Test items for which performance is largely driven by such accent features will not benefit from subtitled exposure.

When we chose the 120 spoken word stimuli, we did so without considering these factors. This decreases the power of our experiments. Indeed, Experiment 1 found only a very small effect when all items were considered: whereas participants in the Absent condition on average had 47% accurate transcriptions, participants in the Concurrent condition on averaged had 50% accurate transcriptions.

To increase the signal-to-noise ratio of our experiments, we are thus interested in determining the test items that benefit from exposure with concurrent subtitles, so that we can ask whether—for those items—*delayed* subtitles also facilitated accuracy during test. We thus conducted a separate norming study using a paradigm identical to Experiment 1 to determine which of the 120 items benefitted the most from the presence of subtitles. We recruited 148 new participants; after applying exclusion criteria identical to Experiment 1, this left 59 participants in the Concurrent condition and 40 in the Absent condition. We then calculated the extent to which exposure to concurrent subtitles improved accuracy compared to exposure without subtitles. Specifically, we repeatedly analyzed the results of the norming study while incrementally removing the items for which subtitle exposure increased performance the least (the analysis we used for these data is the same as reported for Experiment 1 in the result section below). The comparison between the Concurrent vs. Absent subtitles became significant at the *p* < 0.0025 level when items for which subtitle exposure *decreased* performance by 25% or more (a log-odds difference of -1 or less) were excluded (20 items total). These same twenty items were then excluded from the analyses of Experiment 1.

This procedure increased the effect of concurrent subtitles for Experiment 1, compared to the absence of subtitles, from 0.11 to 0.16 log-odds (or 4% improvement, compared to 3% improvement, as described below). We note that this approach makes our analyses anti-conservative with regard to comparisons of the Concurrent condition against any other condition, including any condition with delayed subtitles. However, our approach should not be anti-conservative with regard to the critical comparison of delayed subtitles vs. the absence of subtitles.

### Results

We analyzed transcription accuracy by means of a mixed logit regression [51,52]. Each trial provided one data point. The analysis included three predictors: subtitle condition (coding described below) and two control predictors, the type of audio equipment used (in-ear headphones = 0, over-ear headphones = 1) and the frequency for which participants reported having encountered equally strong accents (never = 0, more than once = 1). The analysis also included the maximal random effect structure justified by the design: by-participant random intercepts and by-item (test word) random intercepts and slopes for subtitle condition.

To establish that we could detect the subtitle benefit in our paradigm, we first Helmert-coded subtitle condition: the first contrast compared Concurrent (1) and Delayed (1) to the Absent condition (-2), while the second contrast compared Concurrent (1) to Delayed (-1; Absent = 0). There was no sign of excessive multi-collinearity (fixed effect correlations *r*s < 0.37). Below, we also present an additional analysis that compares each subtitle condition against the Absent condition.

**Fig 2:**
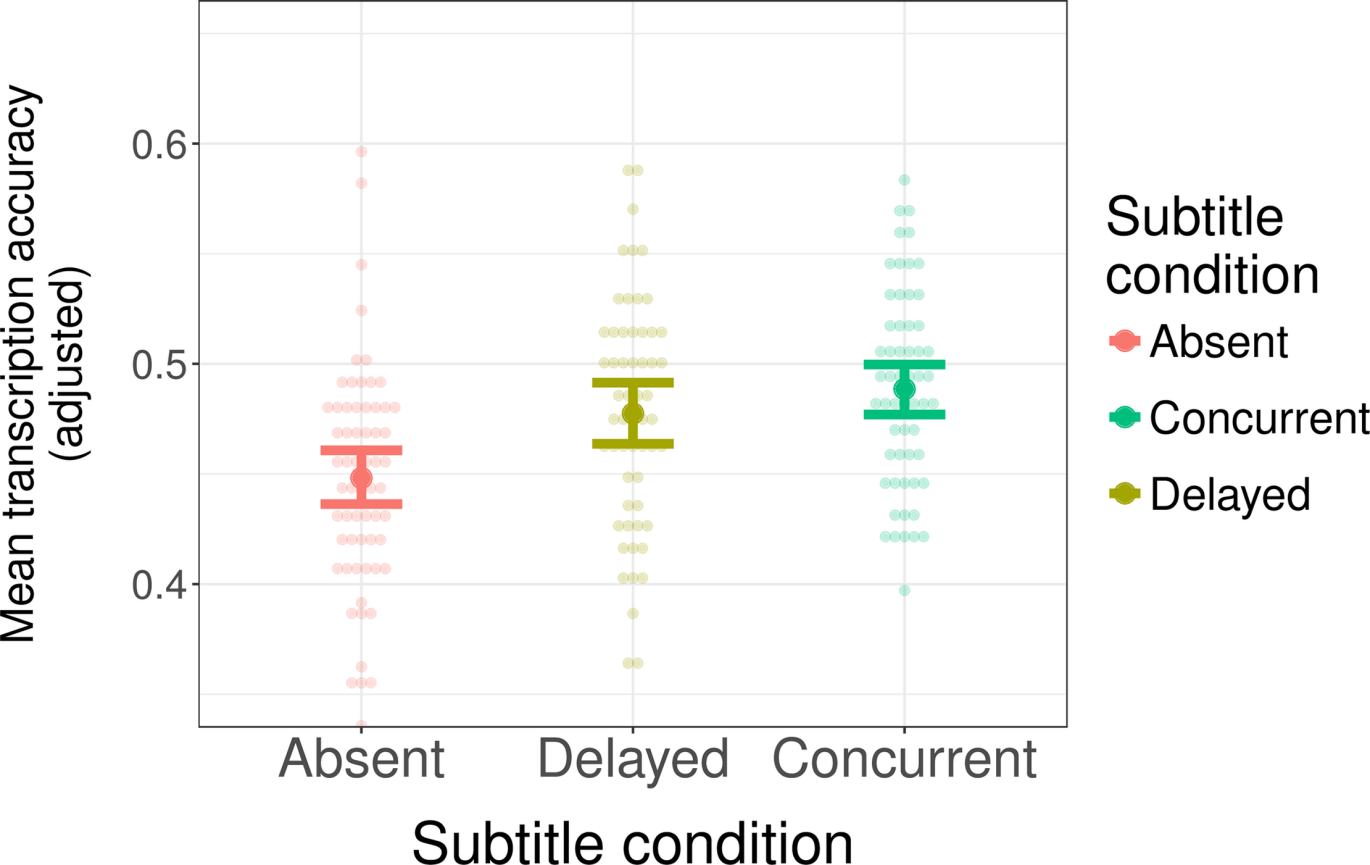
Transcription performance during test in Experiment 1. The values plotted in this graph are adjusted to control for the effects of nuisance variables (see text for details). Dots show individual participants. Error bars show 95% confidence intervals based on non-parametric bootstrap over by-subject means.

There was a significant main effect of subtitle condition on participants' transcription accuracy (Fig 2). As predicted, the presence of subtitles facilitates accent adaptation compared to exposure without subtitles: participants in the Concurrent and Delayed subtitle condition transcribed words more accurately during test than participants in the Absent condition without subtitles with marginal significance ($\hat{\beta }=0.078$, *z* = 2.34, *p* < 0.02). The timing of the subtitles did not seem to matter: transcription accuracy did not significantly differ when subtitles were presented concurrently in exposure, compared to when they were presented immediately afterwards ($\hat{\beta }=0.021$, *z* = 0.339, *p* > 0.7).

The two control variables affected transcription accuracy in the expected direction: accent familiarity significantly improved accuracy during test ($\hat{\beta }=0.305$, *z* = 2.97, *p* < 0.003); over-the-ear headphones (which tend to be of higher quality) lead to numerically improved accuracy compared to in-ear-headphones, but the effect was not significant ($\hat{\beta }=0.042$, *z* = 0.895, *p* > 0.35).

In order to visualize the effects of subtitle condition in a way that discounts the effects of both of these controls, Fig 2 and similar figures below plot *adjusted* transcription accuracy. These adjusted accuracy scores were obtained by using the values predicted by the model for each data point (including the estimated random effects), and holding constant the effect of the control variables at their average values.

To determine the benefits of exposure for the two subtitle conditions independent of one another, a second analysis compared the two conditions for which subtitles were presented (Concurrent and Delayed) to the Absent subtitle condition, using simple contrast coding. I.e., the first contrast being Absent = -1, Delayed = 2, Concurrent = -1, and the second being Absent = -1, Delayed = -1, Concurrent = 2. Transcription accuracy was significantly higher for participants in the Concurrent condition compared to the Absent condition (Concurrent vs. Absent: $\hat{\beta }=0.253$, *z* = 2.12, *p* = 0.034). When subtitles were presented immediately after the offset of the word, participants only transcribed more accurately with marginal significance (Delayed vs. Absent: $\hat{\beta }=0.212$, *z* = 1.88, *p* = 0.060).

### Discussion

First, we find improved transcription accuracy for participants who hear the accented speech presented with subtitles (49%) compared to participants who did not receive subtitles (45%). This extends previous findings that subtitles facilitate the comprehension of regionally accented English for second language learners of English [40]. In this experiment however, we find the same effect for foreign-accented speech in listeners' *native* language.

We also find a marginally significant effect of delayed subtitles compared to the absence of subtitles, suggesting that accent adaptation can—at least in principle—benefit from labeling context that occurs *after* the critical speech input. In fact, we fail to find a significant difference between delayed and concurrent subtitles. This tentatively suggests little or no decay in relevant information over the duration of the word stimulus. Recall that we excluded items that did not show clear effects of concurrent subtitles. Although our exclusion criterion was based on data from another experiment, this approach should *in*flate estimates of the transcription accuracy for the Concurrent condition. If anything, there is thus a bias *towards* a difference higher performance in the Concurrent, compared to the Delayed, condition—biasing against the result we observe here.

This finding is contrary to what would be expected if listeners immediately compress and discard lower-level information. The results of Experiment 1 thus replicate previous findings that listeners can maintain information past word boundaries (e.g., [25–27,29]).

Unlike in previous work on uncertainty maintenance [25–29], the present experiment avoided several properties that would make it easier for participants to determine which aspects of the speech signal to maintain. First, unlike the previous studies which repeated the same one target word pair (e.g. 144 times [28] and 80 times [29]) with the similar preceding contexts (e.g. 24 times each [28] and 80 times [29]), the present experiment never repeated words and did not let participants use preceding context. This makes it less likely that the present results are driven by task-specific strategies uncommon in everyday speech processing. Second, participants had to either maintain information about the *entire* word on each trial, or—if the limits of the systems do not allow this—participants would have had to decide which aspect of the signal to maintain, without easily knowing which aspect would later be most informative.

In the General Discussion, we summarize additional analyses that confirm this interpretation: the overall benefits of delayed subtitles during exposure on transcription accuracy during the test phase originate in benefits across many different types of phonemes. That is, participants did indeed not just attend to a few phones or types of phonological contrasts, providing additional validation for the subtitle paradigm.

## Experiment 2

In Experiment 1, we presented the delayed subtitles immediately after word offset. In order to investigate the limits of maintenance and how sensory decay affects this process, Experiment 2 introduces longer delays between the percept and right context. If rapid sensory decay strongly constrains maintenance, we should expect to see the subtitle facilitation quickly disappear as the amount of time participants need to maintain information increases.

We collected data for four new subtitled conditions: a Concurrent and 0ms Delayed condition (both as in Experiment 1, but with some changes described below), as well as two additional Delayed conditions in which subtitles were displayed after word offset with a lag of 1500 ms or 6000 ms, respectively. These four conditions were compared against the Absent condition from Experiment 1.

### Methods

#### Participants

With the aim to again recruit 60 successful participants for each of the four new between-participant conditions, 300 participants were recruited using Amazon's Mechanical Turk (www.mturk.com). Participants were randomly assigned to one of the four subtitled conditions. Recruitment criteria were identical to Experiment 1.

#### Procedure

**Fig 3:**
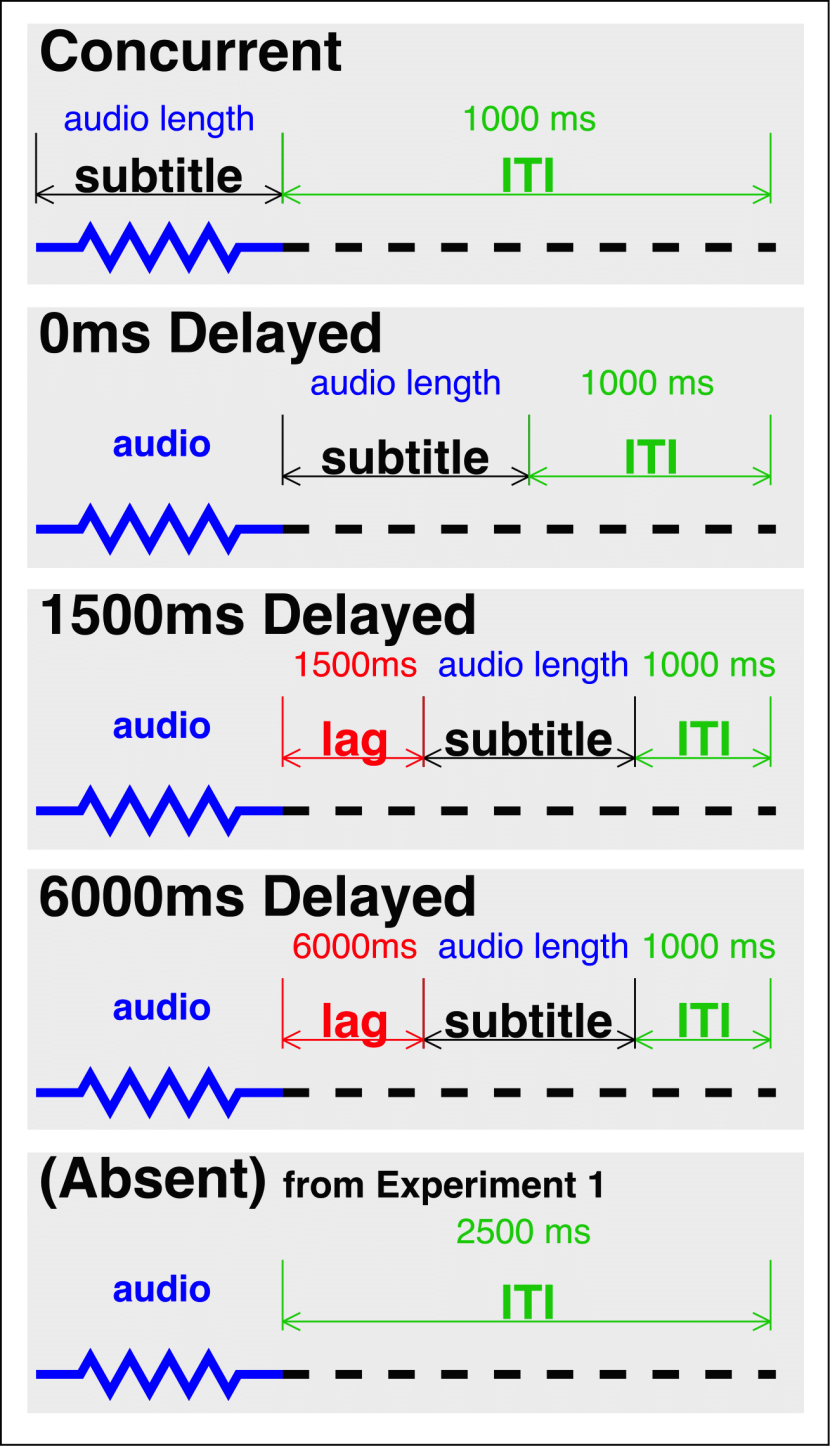
The trial structure of the conditions in Experiment 2 (lengths not to scale). The Absent condition was identical to that of Experiment 1. The analyses presented below compare the four subtitle conditions against the data from the Absent condition of Experiment 1.

The paradigm and stimuli were the same as in Experiment 1, with one small change to the timing of the procedure. In the three Delayed conditions and the Concurrent condition in Experiment 2, the duration of the subtitles was shortened from 1500 ms to the duration of the speech input (which ranged from 379 ms to 848 ms, mean = 586, SD = 86; see Fig 3). Our decision to shorten the subtitle duration was motivated by additional pilot experiments conducted between Experiments 1 and 2, in which we found that shortening the duration of the subtitles actually increased performance, perhaps due to increased task engagement. In Experiment 1, we found no difference between the Concurrent and Delayed condition. By aiming to increase the effect size associated with subtitles, we hoped to also increase our ability to detect differences between the Concurrent and Delayed conditions. The ITI for the four subtitle conditions was set to 1000 ms.

#### Scoring and exclusions

Following the same scoring and exclusion criteria as in Experiment 1, 108 participants in total were excluded from Experiment 2 (Table 2). As with Experiment 1, a majority of these were excluded due to the fact that they were not monolingual. After exclusions there were 62 in Absent condition (taken from Experiment 1), 52 participants in the Concurrent condition, 52 in the 0 ms Delayed, 54 in the 1500 ms Delayed, and 53 in the 6000 ms Delayed condition.

**Table 2:** Participant exclusions in Experiment 2. Some participants were excluded for multiple reasons.

Total participants:	300	100%
Reason for exclusion	n	%
Participant's family likely to include Spanish speakers	17	(6%)
Participant not monolingual	53	(18%)
Participant reported familiarity with strong foreign accents	8	(3%)
Used computer speakers or unknown audio quality	11	(4%)
Client-side computing error	3	(1%)
Failed >4 catch trials (out of 12)	11	(4%)
Outliers in transcription performance	0	(0%)
Participants remaining	176	(70%)

### Results

**Fig 4:**
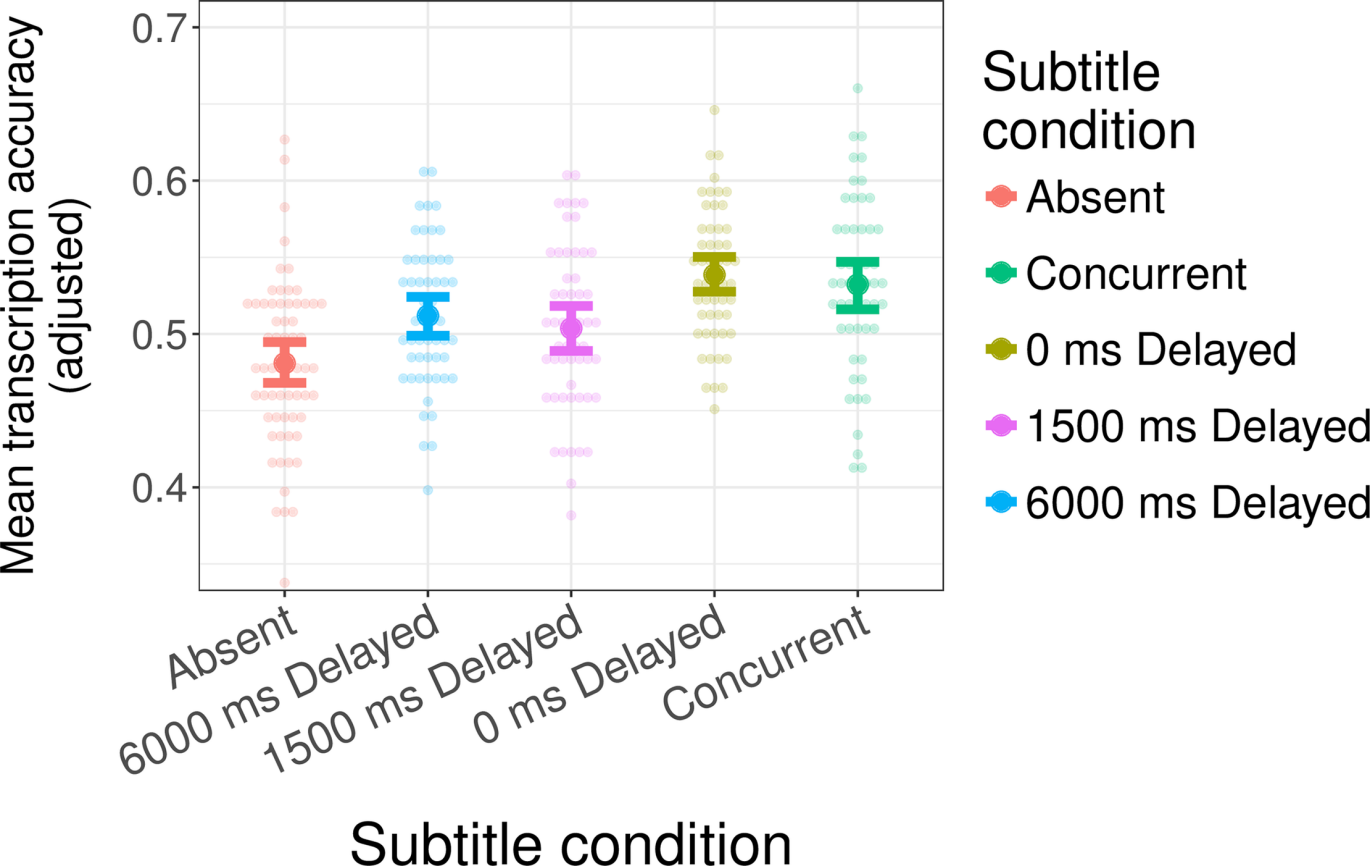
Transcription performance during test in Experiment 2. The values plotted in this graph are adjusted to control for the effects of control variables (see text for details). Dots show individual participants. Error bars show 95% confidence intervals based on non-parametric bootstrap over by-subject means. Despite the general trend of longer subtitle delays conferring reduced subtitle facilitation, even subtitles delayed 6000 ms after the offset of the word significantly improve participants' transcription accuracy compared to the Absent condition.

We follow the same analysis and data visualization approach as in Experiment 1. This includes that we limit our analysis to the same items that we analyzed in Experiment 1. Fig 4 shows the adjusted transcription accuracy for all conditions. We present three analyses to fully assess the effects of the different subtitle conditions.

We first Helmert-coded the different subtitle conditions to compare Absent to everything else (following Experiment 1). Specifically, the contrasts were: 1) Absent = -4, all subtitled conditions = 1; 2) Absent = 0, 6000 ms Delayed = -3, all subtitled conditions with delays >6000 ms = 1; 3) Absent = 0, 6000 ms Delayed = 0, 1500 ms Delayed = -2, all subtitled conditions with delays >1500 ms = 1; 4) 0 ms Delayed = -1, Concurrent = 1, all others = 0. This analysis failed to converge with the full random effect structure, prompting us to remove the correlations among the random effects for the by-item slopes for condition. The comparison of all subtitle conditions against the Absent condition was significant, replicating the benefit of subtitles observed in Experiment 1 ($\hat{\beta }=0.058$, *z* = 3.22, *p* = 0.0013). The remaining Helmert contrasts comparing the subtitle conditions against each other found that participants in the 1500 ms Delayed condition correctly transcribed significantly fewer words, compared to the average accuracy across the 0 ms Delayed and Concurrent conditions ($\hat{\beta }=0.072$, *z* = 2.88, *p* = 0.037). All other comparisons were non-significant (*p*s > 0.3).

The effects of the two control variables numerically replicated those found in Experiment 1. However, this time the effect of accent familiarity did not reach significance ($\hat{\beta }=0.061$, *z* = 0.797, *p* > 0.4), whereas the audio equipment reached marginal significance ($\hat{\beta }=0.067$, *z* = 1.862, *p* < 0.063).

Next, to test *which* subtitle conditions facilitate accent adaptation, we compared each subtitle condition against the Absent condition using simple contrast coding, as in Experiment 1. This analysis converged with the full random effects structure. There were no signs of multi-collinearity (all fixed effect correlations *r*s < 0.50). We found highly significant differences for both Concurrent vs. Absent ($\hat{\beta }=0.358$, *z* = 3.10, *p* < 0.002) and 0ms Delayed vs. Absent ($\hat{\beta }=0.413$, *z* = 3.35, *p* < 0.001), with participants in both of the subtitled conditions achieving higher accuracy than those without subtitles. Numerically, the same trend—facilitation—was also observed for the two remaining conditions with delayed subtitles. However, this effect reached marginal significance only for the 6000ms Delayed condition ($\hat{\beta }=0.212$, *z* = 1.89, *p* < 0.059), and did not reach significance in the 1500 ms Delayed condition ($\hat{\beta }=0.171$, *z* = 1.48, *p* < 0.14).

Finally, to assess whether accent adaptation decreased with increasing delay of the subtitles, we repeated the analysis using backward difference coding (also known as "slide contrast" coding, i.e., 6000 ms Delayed vs. Absent, 1500 ms Delayed vs. 6000 ms Delayed, 0 ms Delayed vs. 1500 ms Delayed, Concurrent vs. 0 ms Delayed). This analysis converged with the full random effects structure. There were no signs of multi-collinearity (all fixed effect correlations *r*s < 0.51). The comparison of 6000 ms Delayed vs. Absent was marginally significant ($\hat{\beta }=0.212$, *z* = 1.89, *p* < 0.059). The comparison of the 0 ms Delayed vs. 1500 ms Delayed condition was significant: with participants in the 0 ms Delayed condition had higher transcription accuracy ($\hat{\beta }=0.242$, *z* = 2.03, *p* = 0.042). All other comparisons were non-significant (*p*s > 0.6).

### Discussion

In Experiment 2, we find further evidence that even when the right-context is delayed until after the offset of the percept, subtitles still facilitate accent adaptation. This suggests that participants are able to maintain information and use it for learning even past word boundaries. There also appear to be marginal improvements in adaptation even after a delay of six seconds between the offset of the percept and the right-context, far longer than previous proposals might have suggested.

We also observe a trend wherein shorter delays have a tendency to improve transcription accuracy more (see Fig 4). There is a significant decrease in transcription accuracy when subtitles (right-context) are delayed 1.5 seconds. This is the first evidence in our paradigm suggesting the limits to such maintenance. Overall, this pattern suggests that while listeners *can* maintain information past word boundaries (tentatively, even after six second delays), longer sensory decay reduces how much relevant information can be maintained for sufficiently long to support accent adaptation.

## General discussion

Some views conceptualize language understanding as "compression", whereby listeners move from more detailed, lower-level representations to more abstract, higher-level ones. However, optimal inferences at a higher-level (e.g., parsing) can require subcategorical information from lower levels (e.g., phonetic properties, [3,53]). Implicit knowledge about subcategorical differences between talkers can also play a crucial role in robust speech perception [54]. How listeners navigate the trade-offs between compressing information and the potential benefits of maintaining information is an open question.

According to the *immediate and complete compression* hypothesis, strong limitations of the cognitive system force us to compress the auditory signal as quickly as possible down to the invariant categories, discarding lower-level, subcategorical information. This view was motivated by early findings such as categorical perception, which quickly dichotomizes input varying on an acoustic continuum into two distinct categories [4]. Although often implicit, this view continues to be influential in research on language processing (for review, see [1]).

An alternative view holds that at least *some* subcategorical information is maintained for longer periods of time, thereby allowing this information to be integrated with subsequent context to affect categorization of earlier percepts. As summarized in the introduction, a number of findings have lent support to this idea. First, auditory information can sometimes persist for relatively long (e.g., pitch for 10 seconds [18], vowel information for three seconds or more [19]). Second, listeners implicit knowledge of words seems to include at least some subcategorical information about talker identity (e.g., [9,10,15]). Third, a number of studies have found that later right-context information can change interpretation of previously encountered percepts in ways that suggest that subcategorical information has been maintained about the earlier percept [30,31,35]. These right-context effects are observed past word boundaries. Indeed, some recent work has found right-context effects up to 6–8 syllables [25–27,29], far beyond what would be expected under immediate and complete compression.

The present study provides further support for the latter view. We find that subtitles during exposure to speech of an unfamiliar accented talker facilitate adaptation, even when the subtitles are delayed. Next, we elaborate on three specific contributions our paradigm makes to research on information maintenance and accent adaptation. We also discuss potential caveats to our interpretation of the results. Following that, we raise considerations for future work within the subtitle paradigm or similar paradigms. We close by discussing the specificity of the information that listeners seem to maintain about the speech input.

### Contributions of the present study

The first two contributions we discuss pertain to the literature on information maintenance, and both contributions originate in the use of the subtitle paradigm. This paradigm has previously been employed in studies on second language processing [40], modulation of early auditory processing [42], and phonetic adaptation [43]. Here, we extended it to the study of information maintenance (see also [42], discussed in more detail below).

The first contribution of the present work is that it allows us to avoid some of the methodological concerns that have been raised about previous work on information maintenance (for a discussion, see also [27]). Most of the paradigms employed in previous work allow participants to limit their attention to specific sounds (e.g., onset plosives [28]) and sometimes even specific locations in the signal (e.g., the onset of the second word [29]). These paradigms also often involve massive repetition of similar stimuli. The subtitle paradigm employed here avoids such repetitions (see also the paradigm in [26]). Additionally, participants cannot easily determine what subcategorical information will be relevant in adapting to an accent. The results of Experiments 1 and 2 thus suggest that listeners can maintain information even when it is unclear which aspects of the signal should be maintained, and where in the signal those aspects might appear. This interpretation of our results is supported by additional analyses of Experiments 1 and 2, which we summarize here briefly (for details, see S1 Appendix).

**Fig 5:**
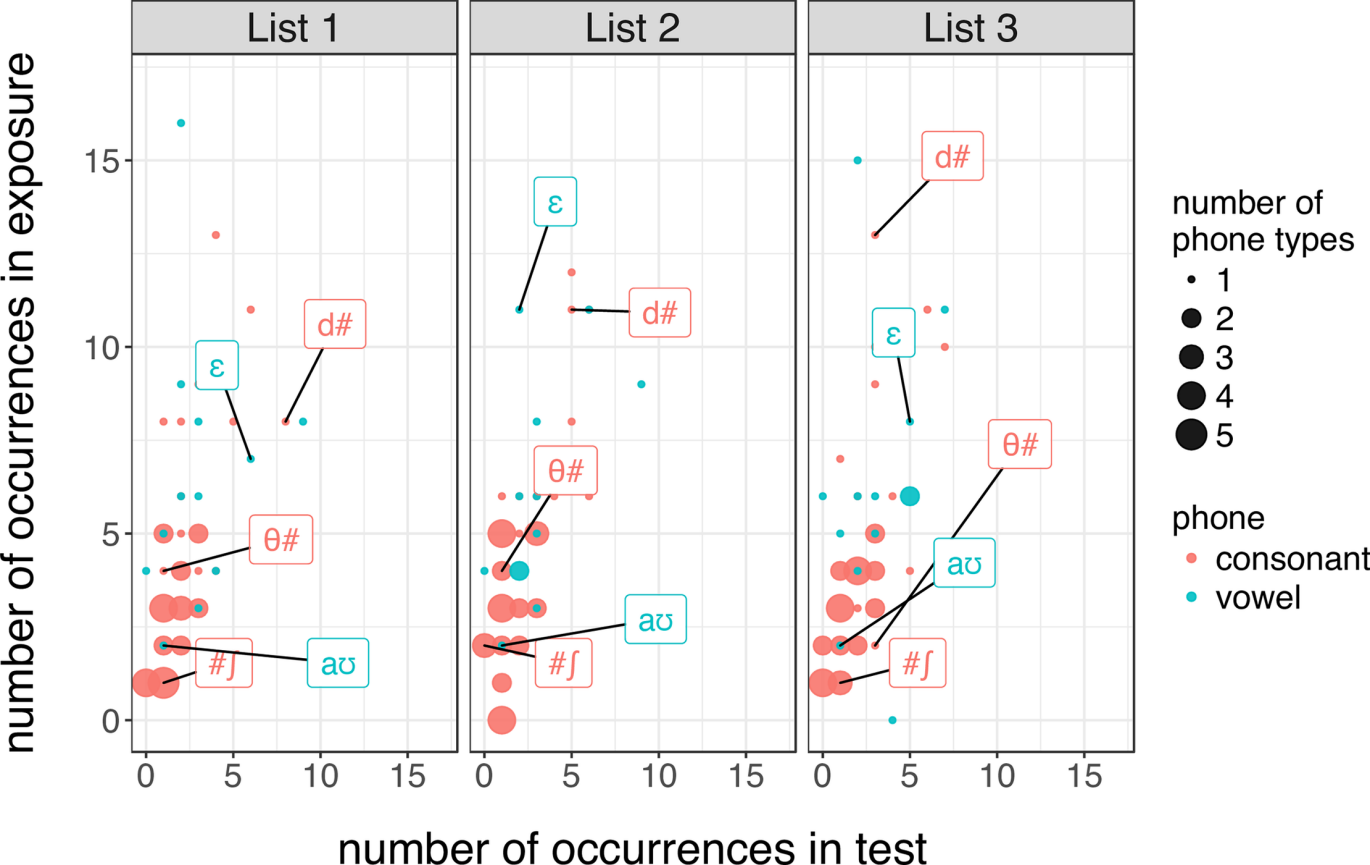
The number of occurrences of each position-specific phone during both the exposure and test phases of Experiments 1 and 2. The dot size indicates the number of phones for that particular set of values. Panels show the three stimuli lists, of which participants were randomly assigned to one. Select phones are labeled in IPA, with # indicating word boundaries (see Fig 6 for the subtitle benefit for the same phones).

*A priori*, one property of our design makes it unlikely that participants attended to only a few phones: each phone occurred only a few times during exposure and test. Fig 5 shows this for phones. This would make it difficult to achieve the observed benefit of subtitles on transcription accuracy by attending to only a small subset of all sound categories during exposure.

**Fig 6:**
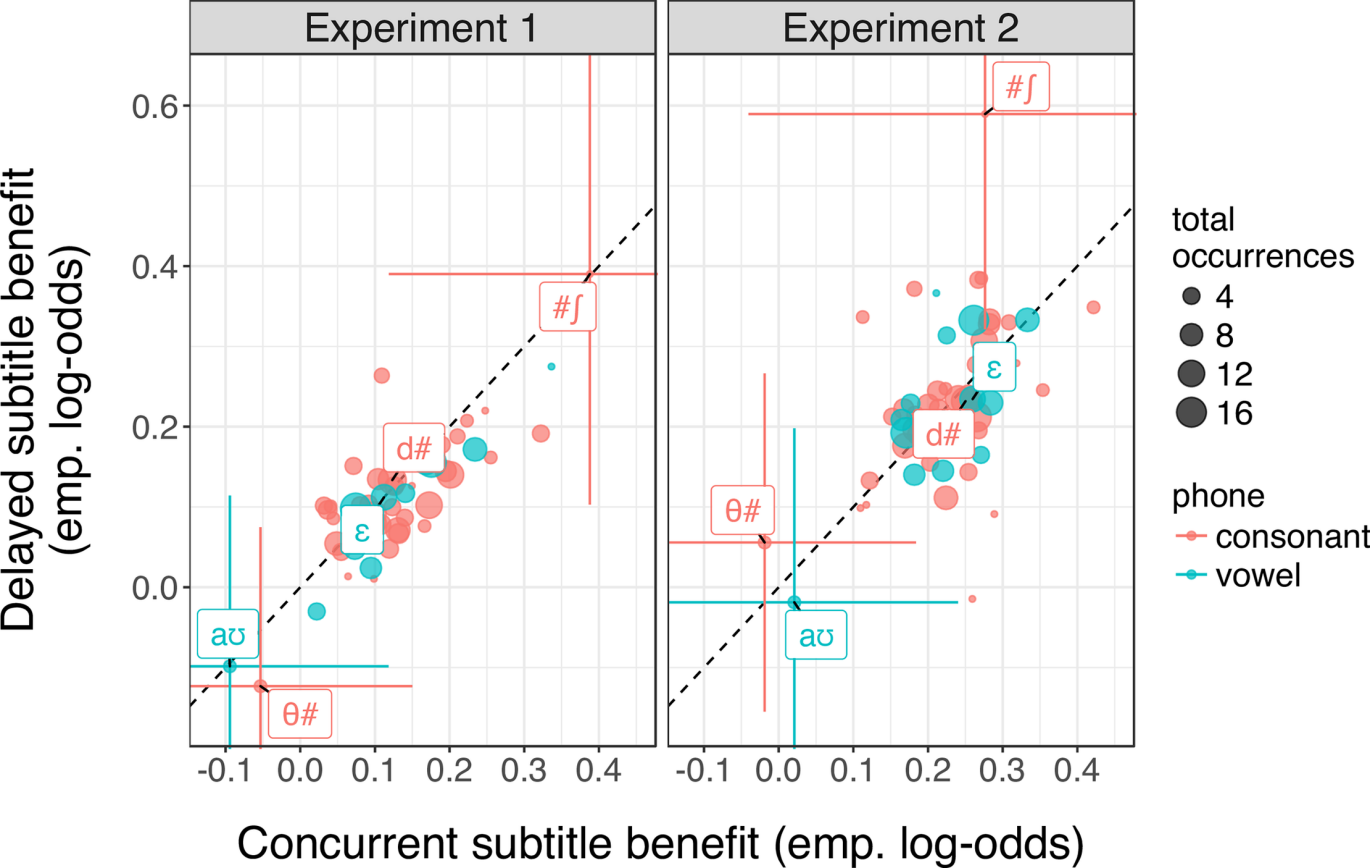
Benefit concurrent (y-axis) and delayed (x-axis) subtitles in exposure for each position-dependent phone during test. The benefit shown here is the difference in transcription accuracy for words containing each phone in the subtitled conditions compared to the Absent condition, in empirical log-odds. Point size indicates the number of instances of each phone during exposure and test. Select phones are labeled with # indicating word boundaries. The realization of word-final /d/, for example, is known to be strongly affected by a Spanish accent [55,56], whereas /ɛ/ is not. To convey a sense of the variability in the data, the 95% confidence intervals for three of the more outlying phones are provided.

Indeed, the benefit of subtitles during exposure is distributed across a large number of phones in our experiment. This is evidenced in Fig 6, where almost all phones show positive benefits of concurrent subtitles. This pattern is expected if participants maintain information about multiple types of phones, validating the motivation for our paradigm.

There is one more property of our data that further supports this interpretation. Previous work suggests that listeners adapt to multiple accented sound categories in parallel in circumstances where contextual information is present concurrently [57]. Our results suggest that this is likely to be equally true for when relevant contextual information occurs after the accented sound (as in our delayed subtitle conditions): we find that the phone-specific benefit of delayed subtitle condition closely resembles the benefit of concurrent subtitles (Fig 6). This suggests that listeners use the same information in both the concurrent and delayed conditions. We take these findings to argue that listeners do not adopt special strategies for delayed subtitles (for corroborating evidence that information maintenance is indeed a default behavior in spoken language understanding, see [27]). Rather, it seems that participants were able to maintain information about multiple sound categories in parallel—despite the high dimensionality of accented speech and as intended by our choice of paradigm—and that this information maintenance facilitated accent adaptation. The subtitle paradigm thus brings us one step closer to understanding the role of subcategorical information maintenance in everyday language processing.

A second contribution of the subtitle paradigm is that it makes it easier for the researcher to control the amount of time between the percept and right-context. This makes it possible to investigate how intervening time affects information maintenance, as we began to do in Experiment 2. In that experiment we found that longer delays resulted in reduced subtitle benefit, suggesting that information maintenance is not without limits, at least not with regard to the information that is required for accent adaptation. A related finding is presented by Sohoglu et al. [42]. Sohoglu and colleagues used fine adjustments to the stimulus-onset asynchronies between degraded speech and subtitles to pinpoint the delay at which the "pop-out effect" of context declined. They found that the perceived clarity of the degraded speech began to decline when subtitles were delayed by ~120 milliseconds past word onset, although significant benefits of subtitles were still observed at delays as large as ~1,600 milliseconds (the longest delay tested).

While the existence of limits is in line with our findings, the rapid decay of the subtitle benefit (on perceptual clarity) seen in [42] seems to be in conflict with the present study. One possible explanation for this seeming conflict lies in the different types of information that subjects might need to maintain for the two different tasks. Sohoglu and colleagues suggest that the effect they examine—whereby information from multiple modalities (i.e., lexical information from vision and auditory information from the degraded speech) is bound together into a single "enhanced" percept—requires *sensory* memory of the audio to be present when the contextual information is integrated (in line with similar results for the integration of other types of visual information, such as video of talkers' lips [58]).

In the current study, we are not measuring the clarity with which participants perceive speech input: we measure how much right context can contribute to whatever information is necessary for *accent adaptation*. It is possible, if not plausible, that such adaptation operates over *phonetic*, rather than *perceptual*, representations, thus requiring maintenance of less fine-grained information (compared to the study by Sohoglu and colleagues). Previous work has found that listeners can maintain phonetic information about isolated segments (e.g., an isolated vowel) for at least 1–3 seconds, whereas memory of pre-phonetic perceptual information seems to decay more quickly (~200-300msecs, [24]). If this asymmetry in ability to maintain phonetic, compared to perceptual, information carries over to whole word recognition, this explains why we observe subtitle effects for delays far longer than those in [42].

The third contribution of the present work is to research on accent adaptation. We find that subtitles facilitate native language accent adaptation with far less exposure than in used in previous work on second language accent learning (approximately 45 seconds of speech input, compared to 30 minutes in [40]). This suggests that a small amount of exposure material is sufficient to investigate maintenance (see also [38,50]). Next, we discuss considerations for future research on information maintenance.

### Considerations for future studies within the subtitle paradigm

The effects of subtitles observed in Experiments 1 and 2 are relatively small. This can become a problem for future research within this paradigm, especially research on the limits of information maintenance: as the benefit of delayed subtitles begins to decline, the predicted effects will fall between a lower bound expected for the absence of subtitles and an upper bound expected for concurrent subtitles. As the difference between these bounds was approximately 4% or less in Experiments 1 and 2, it could be difficult to reliably assess the ranking of different exposure conditions between these upper and lower bounds.

This is, however, not an inherent limitation of the subtitle paradigm. Rather, the small effect sizes might result from our choice of stimuli. By carefully choosing the materials for exposure and test so as to maximize the expected benefit of concurrent subtitles, it should be possible to address this shortcoming of the present study. For example, whereas English has both /ɪ/ (as in “sip”) and /i/ (as in “seep”), Spanish has only one corresponding vowel. In Spanish-accented English, the two English vowels often are pronounced in similar, if not identical, ways [59]. Even perfect adaptation to Spanish-accented English then would only guarantee a 50% chance of differentiating "sip" vs. "seep." Our experiments did not avoid stimuli with such phonological properties (cf. Fig 5). This is likely to have contributed to the relatively small effect of subtitles (compared to contrasts such as voicing in Spanish-accented plosives, which is separable has a one-to-one mapping), as the effectiveness of subtitles depends on the effectiveness of adaptation with regard to the test items. To avoid this issue, future work would benefit from employing items for which larger benefits of adaptation are expected. This in turn might require the use of foreign, dialectal, or regional accented English that have one-to-one, rather than many-to-one category mappings between native and accented pronunciations.

Future studies could also manipulate the amount of speech (or other auditory) material—rather than time—intervening between the percept and the subtitle. This would make it possible to compare to effect of time and the effect of intervening speech on the maintenance of subcategorical speech information. Consider, for example that some previous studies have found effects of right-context for even six to eight syllables past the word boundary [25–27] (but see [28]), while other studies suggest that auditory sensory memory decays relatively rapidly (for a review, see [24]). Understanding how these pieces of evidence relate to each other is important for developing theories of how linguistic information processing: it is possible, for example, that information maintenance is limited more by the amount or type of information that is being maintained than by the time between percept and right-context.

### What information is being maintained?

A big open question for research on information maintenance pertains to the specificity, or type, of the information listeners maintain. There are at least two qualitatively different hypotheses. One possibility is that listeners maintain information that is at or below the level of phonetic information, which we take to be the default assumption of the field. The second hypothesis is that listeners maintain only gradient *uncertainty* about phonological category labels, rather than specific phonetic information. The present experiment was not designed to deliver a decisive answer to this question. It does, however, favor one of the two hypotheses. We elaborate on the two hypotheses and their plausibility given existing evidence—also in an effort to simulate further research on this question.

**Fig 7:**
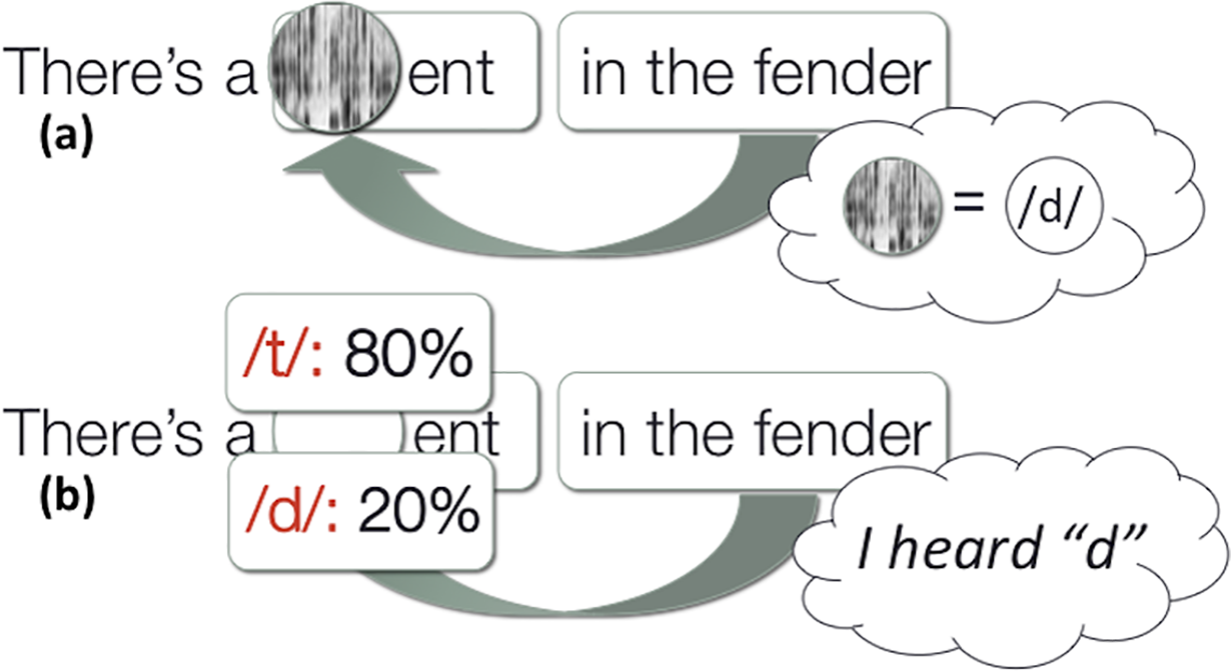
Illustration of two accounts of right-context effects. (a) *Phonetic maintenance*: Listeners maintain some level of subcategorical phonetic information about a phone and integrate this information with later contextual information. (b) *Uncertainty maintenance*: Listeners only maintain the barest amount of subcategorical information to integrate with later context: their relatively certainty about the possible categories. Evidence based on offline categorization tasks employed in previous work is compatible is compatible with either account.

Consider, for example, the type of paradigm employed by [3,28]. Participants heard sentences like "There's a (d/t)ent in the fender," where (d/t) was artificially ambiguous between /d/ and /t/. Under the first hypothesis, listeners maintain subcategorical phonetic information (e.g., VOT information about the ambiguous /t/-/d/ contrast) and then upon receiving right-context (e.g., "fender"), integrate these two sources of information when reporting what they heard (see Fig 7A). But these results are also compatible with the competing hypothesis that listeners maintain only uncertainties about the intended category of the phonemes. Under this hypothesis, after encountering the ambiguous (d/t), a participant might only maintain the information that they were 80% sure it was a /d/ and 20% sure it was a /t/, and after getting the context of "fender", merely combine this uncertainty with the right-context when reporting what they heard (see Fig 7B). This evidential ambiguity is not unique to the paradigm employed by Connine and colleagues: in fact, almost all existing evidence is compatible with either view. Indeed, the theoretical distinction between phonetic and uncertainty maintenance seems to have been raised only recently [25,27,26].

There are a number of studies that speak to the question at hand (although they were not intended to address it). One set of evidence comes from studies on the acquisition of non-native phonemic contrasts during second language (L2) learning. In order to learn non-native phonemic contrasts, listeners must learn the specific ways in which novel phonetic cue combinations relate to L2 phonological categories. L2 contrast-learning studies often train participants to do this by asking them to categorize words or syllables containing the non-native contrast and then giving them feedback on their response. Although many studies present the delayed feedback simultaneously with a repetition of the original audio [60,61], some studies have provided feedback without repeating the audio input. In those studies, the feedback then has a similar right-context function as delayed subtitles in the present study.

For example, in McCandliss et al. [62] native Japanese listeners learned the English /r/-/l/ contrast (which is not present in Japanese) by repeatedly categorizing instances of "rock" vs. "lock." In order to improve, participants must learn to use specific novel phonetic cues to differentiate the unfamiliar phonemic categories. Participants who were given the correct label after each categorization learned to accurately categorize the novel categories more quickly, compared to participants who received no feedback [62]. Under the standard assumption that accuracy improvements in this task reflect learning of novel phonetic cue dimensions (and their relative weighting for categorization), this provides evidence that listeners can maintain *phonetic* information: in order for learning of phonetic cues to benefit from the delayed feedback, it is necessary that *some* phonetic information is maintained until the feedback becomes available.

**Fig 8:**
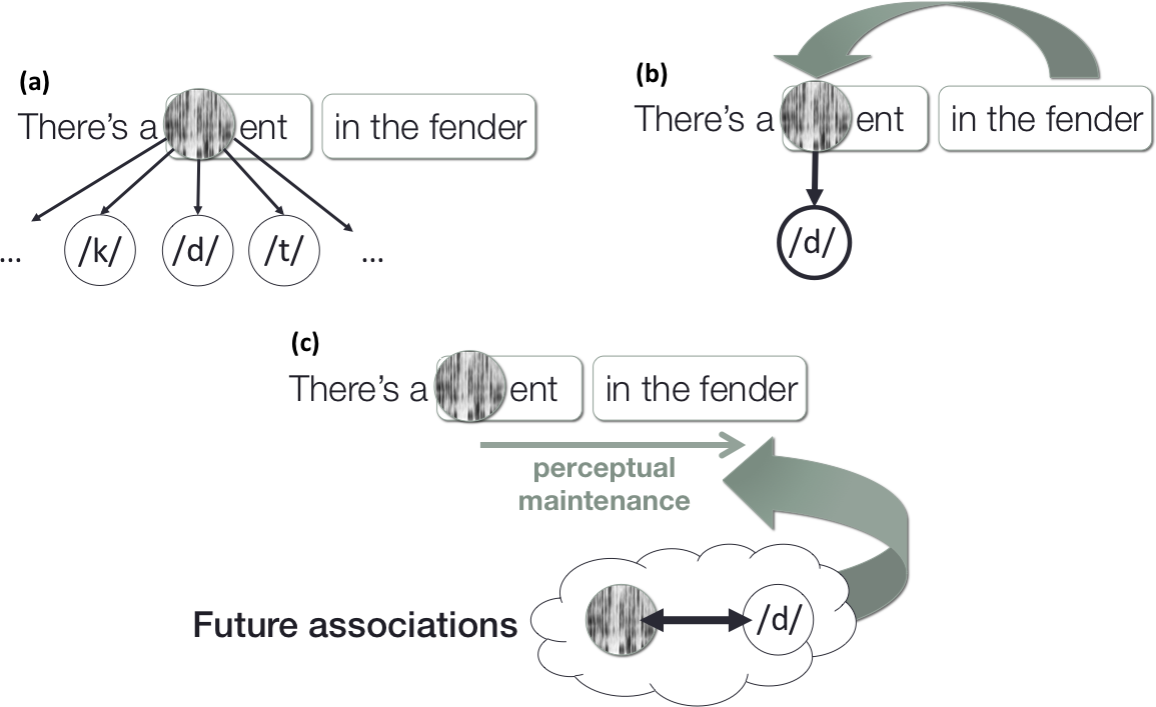
Illustration of how right-contexts might facilitate accent adaptation. (a) When a listener first hears an ambiguous /d/ in "dent," there are multiple likely inferences about the phoneme category. (b) But after hearing the right-context of "fender," /d/ becomes the most likely category for the sound. (c) If listeners learn to connect elements of the percept of the sound to the phoneme category, it would suggest that they maintained some amount of subcategorical information about the percept until they received the right-context.

Phonetic maintenance also provides a natural explanation for own results, following similar logic (see also Fig 8). If listeners can maintain phonetic information until the right-context of the subtitles in the Delayed conditions, they would able to pair the phonetic information with the intended phonological category labels—essentially facilitating learning by access to a teaching signal.

## Conclusions

The idea of quick, if not immediate, compression continues to dominate many researchers' understanding of language processing. There is now a mounting body of work on right-context effects that suggests that this intuition needs to be revisited carefully [3,25–27,29,35] (for a review of earlier works, see [32]). At the same time, subcategorical maintenance must have limits. As it stands, we know relatively little about the nature of these limits and, in particular, how they pertain to everyday language understanding.

Together with the evidence from studies such as [62], the present work extends previous arguments for *why* listeners might want to maintain phonetic information: doing so can facilitate learning and adaptation to different talkers and accents [31,32,33] (and others).

## Supporting information

S1 Appendix Analyzing subtitle benefits and strategies. (DOCX)

S2 Appendix Experimental controls. (DOCX)

S1 Questionnaire Post-test survey questionnaire. (DOCX)
