# Supplementary material for: Maintaining information about speech input during accent adaptation
Source: PLoS One. 2018 Aug 7;13(8):e0199358. doi: 10.1371/journal.pone.0199358 (PMC6080756; doi:10.1371/journal.pone.0199358)
Supplement: S1 Appendix — (DOCX) [file pone.0199358.s001.docx]

# S1 Appendix

## Analyzing subtitle benefits and strategies

In order to determine the plausibility of certain alternative hypotheses, we investigated certain claims about the structure of the items in the experiment as well as the results. In order to calculate the subtitle benefit for the Delayed and Concurrent conditions for each phone in Fig 6, we first assigned the accuracy of each individual trial to each phoneme for that trial, broken down by position (all words were CVC, and we treated word-initial and word-final phones separately). We then measured the benefit as the difference of means in the subtitled conditions compared to the Absent condition for each position-dependent phone in empirical log-odds. Confidence intervals were calculated from the standard errors of the differences between the means of the transcription accuracies for each phone (the subtitled conditions means minus the Absent condition means), which were then multiplied by 1.96 on each side from the differences.
