# Supplementary material for: Maintaining information about speech input during accent adaptation
Source: PLoS One. 2018 Aug 7;13(8):e0199358. doi: 10.1371/journal.pone.0199358 (PMC6080756; doi:10.1371/journal.pone.0199358)
Supplement: S2 Appendix — (DOCX) [file pone.0199358.s002.docx]

# S2 Appendix

## Experimental controls

Our experiments seek to assess effects of different subtitle conditions on accent adaptation. We assess these effects in terms of the proportion of correctly transcribed words during the test phase of the experiments, in which participants in all groups do not see subtitles.

Prior to conducting our experiments, we were concerned that the accuracy of those transcriptions would likely be affected by between-participant differences in the audio equipment and language background. As we manipulate subtitle presentation between participants, differences in audio equipment or language background that are not balanced across our conditions could theoretically confound the results of our experiments.

We thus had participants answer a number of survey questions at the end of the experiment. The full list of questions is provided in the S3 Questionnaire. These questions presented us with a large number of possible predictors to use as controls. Here we describe how we determined the control predictors that were included in the analyses of Experiments 1 and 2—a process that, by necessity, is somewhat explorative.

## Controlling for language experience

Before we started testing the subtitle paradigm, we did not know what type of question would be most informative about participants' language background, including experience with different accents. It is difficult to convey to lay people what constitutes an "accent" or even a particular accent. We were thus worried that participants could vary significantly in their interpretations of our survey questions. We therefore approached participants' language experiences with a variety of questions (listed in the S3 Questionnaire) in order to explore which line of questioning was most useful.

After collecting data from initial tests of the paradigm, we visualized how predictors based off responses to these questions related to general transcription accuracy and ran rudimentary statistical analyses to see how much variance they explained (without taking into account subtitle condition). In general, we found that (as expected) more self-reported exposure to foreign accents correlated with greater performance during test. Most questions turned out to be uninformative about transcription performance. We also found that participants had little to no explicit access to their implicit knowledge of accents. For example, we found that participants who reported more previous experience with the *specific* accent used in the experiment performed *worse* in transcribing the test stimuli. We have replicated this pattern in a number of other experiments on accent perception in the lab that employed the same exit survey questions. One possible explanation for this pattern is that people with *less* accent experience are more likely to overestimate their own experience with any particular accent (e.g., because all accents sound alike to them). Whatever the reason for this pattern, since we could not know that our question was understood correctly, we opted against including this question in our analyses.

We chose a more general question (Question 4 in the S3 Questionnaire) in the post-test survey to control for language background, because this variable had an interpretable pattern and explained a substantial amount of the variance in the dependent variable. This decision was made without knowledge of how this would affect our subtitle results.

## Controlling for audio quality/equipment

We had similar initial concerns about differences in participants' audio quality affecting their performance, and similarly asked an expanded set of post-test questions designed to make sure we could capture these differences. As we expected, during the initial tests of the subtitle paradigm we discovered that participants’ responses about their audio quality were also strong predictors of their performance during test, using the same methods we used for choosing the language experience control predictor.

However, as was the case for some of the questions about participants’ language background, we found that the responses to some questions about the audio equipment did not pattern with transcription accuracy in intuitive ways. For example, participants who reported having "professional" quality audio equipment actually performed worse than those who had merely "excellent" quality equipment.

Again, we included only variables with interpretable patterns that explained significant amounts of variance. Again, this decision was made prior to knowing how this would affect the analysis of subtitle effects.
