# Supplementary material for: Maintaining information about speech input during accent adaptation
Source: PLoS One. 2018 Aug 7;13(8):e0199358. doi: 10.1371/journal.pone.0199358 (PMC6080756; doi:10.1371/journal.pone.0199358)
Supplement: S1 Questionnaire — (DOCX) [file pone.0199358.s003.docx]

# S1 Questionnaire

## Post-test survey questionnaire

After completing the test phase in all experiments, participants were required to answer the following questions. Circular bullet points indicate multiple choice forced answer questions. Square bullet points indicate questions for which participants could select several answers.

For readers' convenience, we also indicate questions and answers that were used in our exclusion criteria or to code the control predictors. These indicators were not provided to participants. Below, questions marked with a single asterisk were used in participant exclusion criteria. For those questions, answers that are underlined led to participants being excluded. Questions that are highlighted were coded as control predictors.

1. Did any of the audio clips jump, stall, or skip during the experiment?
   1.
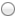
 Yes, very frequently.
   2.
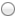
 Yes, a handful of times.
   3.
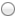
 Yes, once or twice.
   4.
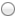
 No, they all played smoothly.
2. *What kind of audio equipment did you use for the experiment? Please be honest. Your response will have no effect on whether you will be paid.
   1.
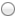
 In-ear headphones
   2.
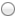
 Over-the-ear headphones
   3.
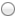
 Laptop Speakers
   4.
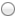
 External Speakers
3. *How does your audio equipment sound when you watch (high quality) music videos on YouTube, watch movies on Netflix, or engage in other similar activities involving audio?
   1.
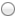
 Poor (most words cannot be understood)
   2.
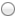
 Okay (many sounds are distorted)
   3.
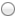
 Good (occasionally some minor distorted sounds)
   4.
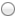
 Excellent (crystal clear with no distorted sounds)
   5.
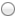
 Professional quality
   6.
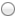
 I don't know or don't do any of the above.
4. *You might have noticed that the talker you heard had an accent. How often do you hear talkers with a similar accent that is EQUALLY STRONG, whether in person or in movies/TV shows etc.?
   1.
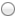
 A few times a day or more.
   2.
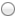
 Perhaps once a day.
   3.
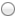
 Perhaps a few times a week.
   4.
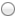
 Perhaps a few times a month.
   5.
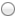
 Perhaps a few times a year.
   6.
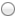
 I don't recall ever hearing this accent before.
5. Please tell us in what context you encounter these talkers (select all that apply)
   1.
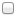
 In my family.
   2.
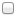
 Among my close friends.
   3.
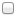
 At work.
   4.
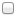
 My own accent sounds like that.
   5.
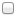
 In movies, shows, etc.
   6.
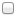
 I never heard this accent.
6. How often do you hear talkers with a similar (but perhaps LESS STRONG) accent?
   1.
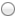
 A few times a day or more.
   2.
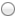
 Perhaps once a day.
   3.
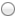
 Perhaps a few times a week.
   4.
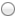
 Perhaps a few times a month.
   5.
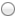
 Perhaps a few times a year.
   6.
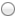
 I don't recall ever hearing this accent before.
7. In what context you encounter these talkers (select all that apply)
   1.
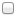
 In my family.
   2.
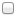
 Among my close friends.
   3.
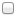
 At work.
   4.
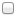
 My own accent sounds like that.
   5.
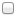
 In movies, shows, etc.
   6.
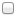
 I never heard this accent.
8. How much did you pay attention during the first task (listening to words). Please answer honestly. This answer will not affect your payment.
   1.
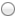
 I tried my best to understand the words the speaker was saying, even though it was hard.
   2.
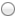
 I lost interest during the experiment and stopped paying close attention to the words.
   3.
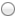
 I did not pay any attention to the words being said and just listened for beeps.
9. How difficult was it for you to understand the talker's accent in the BEGINNING of the experiment?
   1.
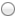
 I understood less than 25% of the words.
   2.
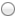
 I understood about half of the words.
   3.
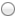
 I understood about 75% of the words.
   4.
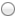
 I understood all but a handful of words.
10. How difficult was it for you to understand the talker's accent at the END of the experiment?
    1.
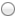
 I understood less than 25% of the words.
    2.
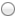
 I understood about half of the words.
    3.
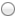
 I understood about 75% of the words.
    4.
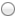
 I understood all but a handful of words.
11. Based on their accent, where do you think the talker is from?
    1. [Free response]
12. *Please check the box that best describes your language background.
    1.
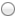
 I speak American English, and NO other languages.
    2.
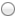
 I speak Spanish and English.
    3.
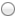
 I speak other languages besides English, but do not speak Spanish.
13. *Please provide us a few more details for your answer above. For example: American English (Native), Italian (Proficient). I lived in Italy for 2 years
    1. [Free response]
14. *(Optional) Enter any other comments (about the experiment, speaker, etc) you might have.
    1. [Free response]

In addition to the post-test survey questions, we also gave participants the option to provide us with demographic information, as required by the human subject review board of the University of Rochester. These questions asked for the following information about participants:

1. Age
2. Sex
3. *Ethnicity
4. Race
